# Porous Ionic Membrane Based Flexible Humidity Sensor and its Multifunctional Applications

**DOI:** 10.1002/advs.201600404

**Published:** 2017-01-24

**Authors:** Tie Li, Lianhui Li, Hongwei Sun, Yan Xu, Xuewen Wang, Hui Luo, Zheng Liu, Ting Zhang

**Affiliations:** ^1^ *i* *i*‐LabSuzhou Institute of Nano‐Tech and Nano‐Bionics (SINANO)Chinese Academy of Sciences (CAS)398 Ruoshui RoadSuzhou215123P. R. China; ^2^ Beijing Information Institute61 Houchangchun RoadBeijing215123P. R. China; ^3^ Centre for Programmed MaterialsSchool of Materials Science and EngineeringNanyang Technological University50 Nanyang Avenue639798SingaporeSingapore

**Keywords:** flexible sensors, humidity sensors, polymer electrolytes, polyvinyl alcohol, porous ionic membranes

## Abstract

A highly flexible porous ionic membrane (PIM) is fabricated from a polyvinyl alcohol/KOH polymer gel electrolyte, showing well‐defined 3D porous structure. The conductance of the PIM changes more than 70 times as the relative humidity (RH) increases from 10.89% to 81.75% with fast and reversible response at room temperature. In addition, the PIM‐based sensor is insensitive to temperature (0–95 °C) and pressure (0–6.8 kPa) change, which indicates that it can be used as highly selective flexible humidity sensor. A noncontact switch system containing PIM‐based sensor is assembled, and results show that the switch responds favorably to RH change caused by an approaching finger. Moreover, an attachable smart label using PIM‐based sensor is explored to measure the water contents of human skin, which shows a great linear relationship between the sensitivity of the sensor and the facial water contents measured by a commercial reference device.

## 1 Introduction

Recently, flexible electronic devices have gotten increasing attentions for potential applications in individual medical care, health assessment, sports monitoring, etc.^1^, ^2^, ^3^ Especially, the rapid advances in the design of various flexible sensors have tremendously broadened the scope of flexible electronics to new classes of soft electronic systems.^4^, ^5^ For example, the flexible pressure and temperature sensors can be proposed for deriving various applications including the skin‐like electronics (E‐Skin), robotics human–machine interfaces, and biomedical applications.^6^, ^7^, ^8^, ^9^, ^10^ On the other hand, flexible humidity sensors were rarely reported, though humidity is one of most‐intensively measured variables in our daily lives, including comfortable living environment, medical facilities, and body health information.^11^ So far, different kinds of sensing materials, such as carbon nanomaterials, metal nanowires, and porous ceramics have been explored to enhance the sensing performances of humidity sensor,^12^, ^13^ but seldom can satisfy the requirements of a qualified highly sensitive and selective sensor.^14^ For example, heating equipment and temperature compensation are usually required during the dehydration process for ceramic and metal oxide based humidity sensors, and the complex and time‐consuming preparation technologies must be overcome for field effect transistor based sensors.^15^, ^16^, ^17^, ^18^ Therefore, new materials and fabrication methods are urgently needed for high‐performance humidity sensing materials, which are (1) suitable for detecting across a wide range of humidity conditions, (2) highly sensitive and selective, and (3) with rapid response times.

At present, the polymer‐based films that possesses high affinity for water resulting from the inherent hydrophilic functional groups are considered as a feasible potential for humidity sensor applications;^19^ and their low‐cost process, high mechanical stability, and flexibility are vital for future applications in wearable/attachable devices.^20^, ^21^ Especially among them, polyvinyl alcohol (PVA) is reported that its volume can be over ten times bigger than the initial state after absorbance of water^22^; and it is known that PVA/KOH polymer gel‐electrolyte is commonly used as the ionic conducting layer in all‐solid‐state flexible supercapacitors because of its great conductivity and mechanical stability. However, to the best of our knowledge, there is little reference has been reported about the PVA/KOH film based flexible humidity sensor due to the low porosity and high intrinsic resistance usually leading to poor sensitivities to relative humidity (RH).^23^

Porous film structure can boost the penetration and diffusion of moisture, and the homogeneous resistance distribution can enhance the transfer of protons through an uniform pattern.^24^, ^25^, ^26^ Here, we developed a flexible and 3D porous ionic membrane (PIM) based high performance flexible humidity sensor. The highly conductive PIM was fabricated by facile spontaneously evaporating the extra water in above‐mentioned gel‐electrolyte, and the humidity sensing results display that its electro‐conductivity changed more than 70 times as the RH increased from 10.89% to 81.75% at room temperature. The response mechanism are carefully discussed which due to the enhanced physical adsorption benefiting from the high surface‐to‐volume ratio and uniform pore distribution. The PIM sensor had fast and reversible response and was insensitive to temperature and pressure change. Two smart systems were constructed to demonstrate the multifunctional applications of the flexible PIM humidity sensors. One is a noncontact smart switch, which was able to recognize an approaching finger by detecting the humidity change on the fingertip with different distances, and the other is an attachable flexible device to predict the water contents of human skin. These applications demonstrated that the novel flexible PIM humidity sensor has great potential in flexible wearable electronic devices.

## 2 Results and Discussion

**Figure 1:**
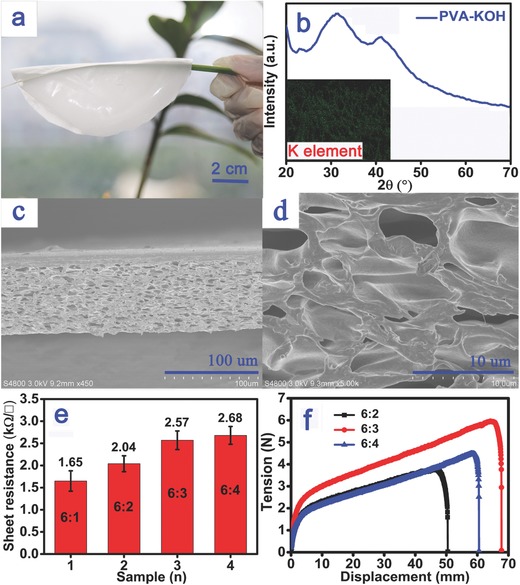
a) A photograph of the as‐prepared PIM sample. b) XRD pattern and c,d) typical SEM images of the PIM sample with a PVA:KOH ratio of 6:3; the inset is an image of the distribution of potassium in the film. e) The sheet resistances and f) the mechanical properties of various PIM products prepared by the same process with thicknesses of ≈70 µm.

An as‐prepared highly flexible PIM sample (diameter: 10 cm) with PVA:KOH mass ratio of 6:3 was shown in **Figure** **1**a (the ultralight PIM was picked up by a leaf stem). The PIM can be produced on a large‐scale and at low‐cost with the highly transparent and homogeneous PVA/KOH electrolyte precursor solution, which was readily prepared by mixing PVA and KOH in deionized water at room temperature (Figure S1a, Supporting Information). The X‐ray diffraction (XRD) analysis (Figure 1b) shows there was a weak but broad peak for the PIM amorphous structure; while no peak was observed for the KOH crystal.^20^, ^21^, ^22^, ^23^ The information in this plot, when considered along with the image of the uniform distribution of potassium in the PIM (the insert in Figure 1b), implies that the KOH dissolved in the PVA solution to form the film without any redundant crystallization after cooling, which is consistent with Figure 1a. The scanning electron microscopic (SEM) images in Figure 1c,d show the detailed architecture for the interior of the PIM. The interior of the as‐synthesized film was characterized as abundance of well‐defined 3D porous and microcavernous structures with an average pore size of about 10–12 µm, which was caused by evaporating water during the drying process. The results also did not show any grain that formed on the smooth pore surface, which also illustrated the uniformity of the polymer electrolyte solution and the as‐prepared PIM.^27^ Further, the sheet resistances of the samples were checked using four probe measurements. The results in Figure 1e show that the average sheet resistance values of four wafer‐sized PIM samples (6 in.), which were uniformly distributed and increased from 1.65 ± 0.23 to 2.68 ± 0.2 KΩ**γ** ^−1^ as the proportions of PVA and KOH increased from 6:1 to 6:4. And the mechanical properties of the obtained PIM samples were also tested in a stretching apparatus (Instron 5900) as shown in Figure 1f and Figure S1b in the Supporting Information, displaying that the PIM with a mass ratio of PVA/KOH = 6:4 had the best tensile force as high as 5.97 N (and its corresponding tensile strength is calculated as 9.2 Mpa according to the National Standard of Tensile Testing (GBT228‐2002)) and breaking strain (383.6%). The high strength and uniform electrical performances are the result of the favorable architectural features described above, such as the homogeneous dissolution and porous microstructure.

**Figure 2:**
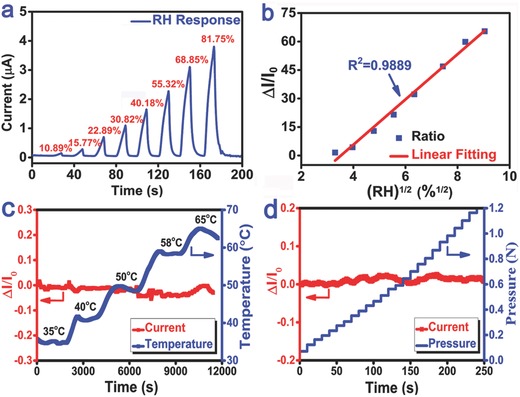
a) Real‐time response of the PIM‐based sensor to RH ranging from 10.89% to 81.75%. The test conditions were adjusted by mixing moist and dry air inside the measurement chamber (0.7 cm^3^) under different flow rates. Data were collected every 10 s. b) Sensitivity (Δ*I*/*I*_0_) of the flexible device versus the square root of different RH. The sensitivity curves of the humidity sensor for temperature and pressure are shown in (c,d). In (c), the temperature was changed from 35 to 65 °C and humidity was maintained at the same level; in (d), the pressure on the sensor was increased step‐wise from 0 to 1.2 N. The PIM sample had a PVA:KOH mass ratio of 6:3 and was ≈70 µm thick.

The humidity response characteristics of this PIM‐based flexible sensor (with a mass ratio of 6:3, and about 70 µm thick) were evaluated by measuring its electrical current under different RH conditions at room temperature.^28^ The sensitivity of the sensor was determined with the following equation

(1) $$\mathrm{Sensitivity}=\Delta I/I_{0}\;\times \;100\%\;=\;\left(I\;-\;I_{0}\right)/I_{0}\;\times \;100\%$$

where *I* and *I*_0_ are the electrical currents at a given and initial humidity level, respectively (the test method is described in detail in the Experimental Section). The real‐time dynamic response curve in **Figure** **2**a indicates that, as RH increased, the current also increased dramatically, which was related to the increase in water physical adsorption and condensation.^29^ With RH increased from 10.89% to 81.75% (the widest RH range can be achieved by the bubbling method described in Experimental Section.), the sensor's current displayed more than 70 times change and showed great linearity according to Langmuir adsorption isotherm model (Figure 2b).^30^ And the sensing performance in Figure S2a,b in the Supporting Information reveals that this PIM‐based sensor also had great response capability as low as 6.42% RH and as high as 93.54% RH. (saturated LiBr and KNO_3_ solutions were sealed in glass bottles at 25 °C to obtain constant RH conditions of 6.3%, and 95%, respectively). Moreover, the stability of the humidity sensor has been tested under the same detected humidity condition (RH: 81.25%) in 4 d after the sample exposed in ambient environment, and the result in Figure S3 in the Supporting Information shows that the four humidity sensing tests of the PIM sensor are basically consistent (sensitivity ±2.5%) with each other, indicating its performances are stable under the ambient condition of CO_2_ and other gases existed. The 3D porous structure of PIM with a lot of active sites can ensure high sensitivity, good stability, and a wide range of dynamic response, on which water molecules can connect with the sensitive material as the capillary condensation increases at different levels.^31^ The response and recovery times are also significant factors for estimating the sensing properties of a humidity sensor. As shown in Figure S2c,d in the Supporting Information, the PIM‐based sensor responded very fast to (about 0.4 s), and recovered quickly after (about 2.6 s), sudden changes in the RH between 47.25% and 80.34% (the ambient RH value was about 47.25%). The response and recovery times are much faster than those previously reported humidity sensors based on other nanomaterials, such as graphene oxide and commercialized humidity sensors.^11^, ^12^, ^13^, ^14^, ^15^, ^16^, ^17^, ^18^, ^19^, ^25^, ^26^, ^27^, ^28^, ^29^, ^30^, ^31^ We can therefore conclude that the polymer electrolyte film serves as a highly conductive electrical pathway that enables rapid migration of electrons and a fast response time, similar to its role in the field of all‐solid‐state flexible supercapacitors.

Previous studies have demonstrated that temperature usually has great impact to the performances of a humidity sensor. For example, carbon nanotube (CNT)‐based sensors are very sensitive to temperature because the binding and activation energies between water molecules and CNT are highly dependent on temperature.^11^, ^12^ Here, the influence of environmental temperature on the humidity sensing of the PIM‐based sensor was measured at temperatures ranging from 35 to 65 °C, which is the most applied temperature condition for the flexible wearable/attachable electronics (wider range from 0 to 95 °C was displayed in Figure S2e in the Supporting Information) under a constant humidity (45% RH) in sealed condition (Figure 2c). The results indicate that there was very little current change of the sensor. The insensitivity to temperature change is due to the ion migration ability of the PIM can be maintained at a stable level and will not alter with temperature change in certain range under an all‐solid‐state. The sensor's response to pressure was also tested a constant ambient humidity (75% RH) condition, in which an pounding head was held perpendicular to the PIM‐based sensor and stroked the sensor with incrementally pressures ranging from 0 to 6.8 kPa. The results show that there were no visible physical damages in the porous morphology of the PIM (Figure S4, Supporting Information); moreover, the sensor's current kept consistent regardless of how the pressure change was applied to the sensor, as shown in Figure 2d. The insensitivity to temperature and pressure is very beneficial to the PIM‐based humidity sensor since the integrated flexible system can be simplified and with light weight, because packaging or compensation circuits for temperature or pressure are not really required.

The humidity performances of PIMs with various mass ratios and thicknesses were systematically investigated. The sensitivities of the four as‐prepared products were determined at PVA:KOH mass ratios of 6:0, 6:1, 6:2, 6:3, and 6:4 (Figure S5a, Supporting Information). The results infer that the ratios can boost the moisture adsorption process as the conductive ability of PIM changes with additions of the KOH (Figure 1f). However, when the ratio reached 6:4, the sensitivity reduced due to larger amount of KOH separating crystals out during the cooling‐down process (Figure S6, Supporting Information), which is caused by the quantity of KOH dissolved in the PVA solution exceeds its maximum dissolving capacity. On the other hand, the thickness of the PIM sensing membrane also plays a crucial role in determining differences in the sensing capabilities. Here, when the thickness range from 16.2 to 125 µm in our experiment, the humidity sensitivity of the PIM‐based sensors first increased to 500% and then decreased as the thickness increased (Figure S5b, Supporting Information). As shown in Figure S7 in the Supporting Information, this may be attributed to the improvement of the inner porous structure was occurred along with the thickness increasing until the superficial through‐hole structure for moisture entering was blocked by the excess precursor. Hence, this comparison of the PIM properties for different proportions of precursors showed that its performance was optimal when the PVA:KOH mass ratio was 6:3 and the thickness was about 70 µm.

**Figure 3:**
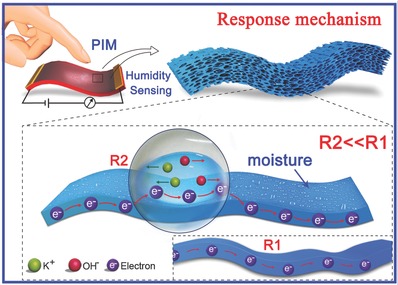
The humidity‐sensing mechanism of the PIM‐based sensor.

The basic humidity‐sensing response mechanism of the PIM‐based sensor can be explained as follows (as the schematic shown in **Figure** **3**): A family of models considers the partition of water uptake into two species that are commonly called “bonded” (that create low energy bonds with hydrophilic sites) and “unbounded” (that transported in free volume) water molecules. Initially, chemical adsorption of water molecules occurs. The alcoholic hydroxyl group (*X_n_*—OH) in the PVA molecular chain is active in this process, the water molecule will reacted with the hydrophilic sites producing “bonded” water based on the diffusion of free volume, water/polymer interaction, resulting in the inner porous surface of the PIM is covered by a monolayer of water molecules.^32^ After the first chemically adsorbed layer forms, the process of physical adsorption of water molecules continues by two adjacent hydroxyl groups attached to the corresponding water molecules.^33^, ^34^ Higher humidity levels give rise to a higher number of physically adsorbed layers, in line with the Grotthuss chain reaction,^35^, ^36^ resulting in a gradual process of capillary condensation of water molecules, consistent with Kelvin's equation.^37^, ^38^ Importantly, the potassium hydroxide has a higher ability to dissolve in water than in PVA, so, during the adsorption process, some potassium and hydroxide ions are transferred from the PVA to the adsorbed moisture and form a liquid electrolyte; the number of transferred electrons in the adsorbed moisture increases dramatically as a result of the directional migration of potassium and hydroxide ions in the electric field, so that the resistance value of the PIM decreases sharply in humid environments (*R*2 << *R*1); The electrical impedance spectroscopy data of the sensor from dry air to 61.42% RH for several cycles were displayed in Figure S8 in the Supporting Information, which were also varied along with the RH change, demonstrating the excellent ionic conduction of the sensor. The increase in conductance is related to the combined influence of chemical and physical adsorption processes, but the chemical process proceeds more slowly than the physical process, so the increased conductance is mainly caused by an increase in physical adsorption.^39^, ^40^ And in desorption process, along with the “unbounded” water transported out the PIM material, the “bounded” water was also divorced from the hydrophilic sites. Though the change of the solubility was appeared as decreasing linearly with the time, the changing slope of desorption was rather lower than that of adsorption.^32^ This may be the reason why the recovery time (2.6 s) of the PIM‐based sensor is rather slower than the response time (0.4 s). Moreover, above unique temperature and pressure insensitive behaviors can be attributed to the electric variation of this membrane mainly based on the migration of its internal conductive ion, which can be stabilized in the polymer mixture and scarcely transferred by the external temperature and pressure change.^21^, ^22^, ^23^, ^24^, ^25^, ^26^, ^41^

**Figure 4:**
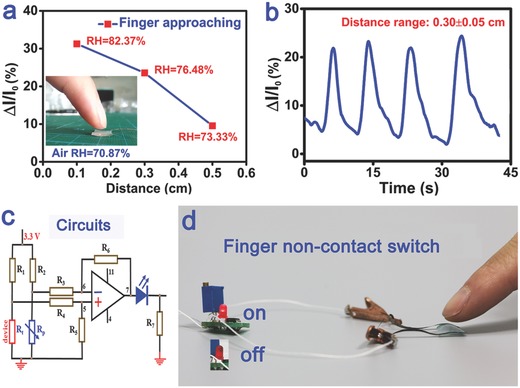
a) Measurements of the PIM‐based sensor at different distances between the finger and the PIM (air RH of 70.87%); the inset shows the scene of a finger approaching the PIM. b) The repeatability of the noncontact PIM‐based sensor for 0.3 ± 0.05 cm with four circles under air RH of 76.48%. c) The configuration of the noncontact switch circuit system; the sensor resistance was *R_t_* and the reference resistance was *R_p_*. d) The photograph of a finger noncontact humidity switch system, and it will display the finger approaching by the on–off and degrees of brightness of a LED light. The PIM (≈70 µm thick) was obtained at a mass ratio of PVA:KOH = 6:3.

Because of the high sensing performance of the PIM‐based humidity sensor, the flexible sensor was can be used to multifunctional applications. For example, it can be applied to detect the moisture around fingertips. A commercial RH sensor was used to calibrate the RH values of the fingertips. The results in **Figure** **4**a show that the current of the sensor suddenly increased when the finger was approaching the PIM, and the variations in the RH sensitivity reached 10%, 22%, and 30% at distances of about 0.5, 0.3, and 0.1 cm, respectively (the surrounding RH was 70.87%). Repeatability is a desirable property for practical applications of a humidity sensor as shown in Figure 4b, indicating that the noncontact sensor has great reproducibility with stable signal outputs. Further, We constructed a noncontact smart switch system in which this noncontact sensor (marked as *R_t_* in Figure 4c) was integrated with external amplifying circuits. As shown in Figure 4d, the variation of *R_t_* caused by the local RH changes with an approaching finger, inducing corresponding changes in the output current. At the same time, the amplifying circuit transferred the changes in the electrical current between *R_t_* and *R_p_* (reference resistance) into voltage shifts, so that the amplified result was automatically displayed by the on–off and degrees of brightness of a light‐emitting diode (LED) light, as shown in the video (SI‐Video‐Finger Noncontact Response, Supporting Information). And the sensitivity of the noncontact devices can be optimized through dosing water‐retention material and reducing the number of external circuits.

**Figure 5:**
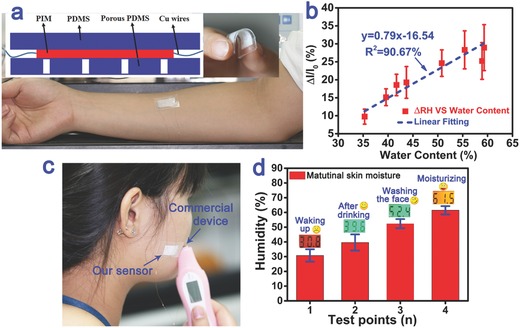
a) The PIM‐based sensor was fixed onto the human skin to assess the humidity changes of different people at the same level; the inset part shows this sensor was assembled with PDMS film, porous PDMS film and the PIM as sandwich structure; and the inset photograph displays its excellent flexibility. b) The linear relationship between the sensitivity of the sensor and the water content of the human skin according to the testing conducted in (a). c) Our sensor was applied to check the water content of the facial skin in the morning; a commercial device based on metal electrode was compared as reference. d) The variation of the facial water content at different measuring time points when waking up (5 min later), drinking (30 min later), washing the face (5 min later), and using moisturizer (5 min later), respectively. The columnar data results were obtained from our PIM‐based sensor and the digital results were read from the commercial device. The PIM sample (≈70 µm thick) was obtained at a mass ratio of PVA:KOH = 6:3.

The water content of human skin is a crucial parameter to assess human health and skin conditions, especially useful to remind people to use skin care products. Here, for the first time, we designed an attachable smart label attempted to establish a function relationship between the sensitivity of our PIM‐based sensor and the water content of human skin. To achieve this purpose, the PIM was attached to a porous polydimethylsiloxane (PDMS) film substrate, which can ensure the humidity of skin can effectively interact with the PIM without directly contacting with the skin (Figure S9, Supporting Information). And another nonporous PDMS film was used as outer layer packaging to prevent the interference from the RH in surrounding environment (**Figure** **5**a). A high‐precision commercial water measurement instrument (Sensirion EX‐H4) was used to measure the actual water contents of the test subject's skin. As shown in Figure 5b, there was a good linear relation (*R* ^2^ = 90.67%) between the sensitivity of the PIM‐based sensor and the actual water contents data measured by Sensirion EX‐H4. Using this relationship, the real‐time water content of a person's skin can be accurately predicted with this smart label. For example, our PIM‐based sensor was applied to check the water contents of the facial skin in the morning, and a commercial device (Skin Moisture Detection Device SQNO.1) was set as a reference (Figure 5c). The measurements were selected at the time points after waking up (5 min later), drinking (30 min later), washing the face (5 min later) and using moisturizer (5 min later), respectively. As shown in Figure 5d, the result demonstrates that our sensor can offer the real‐time solution to detect the water content variation of human skin, such as 30.8% after waking‐up, 39.6% after drinking, 52.4% after washing the face and 61.5% after using moisturizer.

## 3 Conclusion

A PIM with well‐defined 3D porous structures was prepared by naturally evaporating the PVA/KOH polymer gel electrolyte, and its humidity sensing properties showed that a PVA/KOH mass ratio of 6:3 and thickness of about 70 µm were optimal process. Characterizations reveal that the conductivity of PIM‐based sensor changed more than 70 times as the RH value increased from 10.89% to 81.75%, with rapid response (0.4 s) and recovery times (2.6 s). It is noteworthy that this sensor was insensitive to temperature and pressure change, indicating that it is a successful candidate for highly selective flexible humidity sensors. Furthermore, the applications of the PIM‐based sensors were successfully demonstrated. A noncontact smart switch can respond rapidly to an approaching finger, and an attachable smart label was developed to real‐time access the water content of human skin. These performances will offer the tremendous opportunity to apply the PIM‐based sensors to multifunctional intelligent wearable systems.

## 4 Experimental Section

*Preparation of the PIM Sample and the PIM‐Based Humidity Sensor*: The PVA/KOH porous ionic membrane was fabricated using a solution‐casting process at ambient conditions. A PVA solution was produced by mixing PVA (6 g) in deionized water (115 mL) and stirring continuously at 85 °C for about 2 h. Then, a freshly prepared cold aqueous solution (5 mL) of KOH (3 g) was added drop‐wise to the prepared PVA stock solution. No other additives were used to promote the reaction, thereby avoiding the effects of associates or impurities. The prepared PVA/KOH polymer electrolyte mixture was then cast on a glass plate and dried at room temperature, and, once dry, the PIM membrane was peeled off the glass plate. The amount of KOH in the electrolyte solution ranged from 1 to 4 g, and the thickness of the PIM samples, measured by SEM, ranged from 16 to 125 µm. Finally, the PIM‐based humidity sensor was produced by fixing a flexible electrode on the surface of the cutting membrane with silver paste.

*Humidity Sensing Test for the PIM‐Based Sensor*: Figure S10 in the Supporting Information shows the measurement set‐up. Wet (water‐saturated) and dry air were mixed under different mass ratios to obtain various stable RH conditions ranging from 10.89% to 81.75%. The air was water‐saturated by bubbling dry air through saturated salt solutions sealed in a flask. The RH value of the test environment was adjusted by mixing moist and dry air inside the measurement chamber (0.7 cm^3^) at different flow rates, controlled by flow controllers. The actual humidity values were confirmed with a high‐accuracy general RH sensor placed inside the measurement chamber (Sensirion EX‐H4). Changes in the current were measured with a digital source meter (Keithley 2400). The data acquisition step was 10 s, based on which it took over 15 s to fill/evacuate the testing chamber.

*Assessing the Detective Limits and Response and Recovery Times*: Various constant RH conditions were achieved by humidity bottles containing saturated solutions of different metal salts. LiBr, LiCl, MgCl_2_, Mg(NO_3_)_2_, NaCl, KCl, and KNO_3_ were sealed in glass bottles at 25 °C to obtain RH conditions of 6.3%, 11%, 33%, 54%, 75%, 85%, and 95%, respectively, thereby facilitating sudden changes in the environmental RH by transferring the sensor between the dry air and the achieved constant RH. The true value was checked by a commercial sensor (Sensirion EX‐H4). During detective limits and times testing, the PIM sensor was transferred between the RH vessels as quickly as possible to lower the risk of measurement error.

## Supporting information

As a service to our authors and readers, this journal provides supporting information supplied by the authors. Such materials are peer reviewed and may be re‐organized for online delivery, but are not copy‐edited or typeset. Technical support issues arising from supporting information (other than missing files) should be addressed to the authors.

Supplementary

Supplementary
