# Supplementary material for: Porous Ionic Membrane Based Flexible Humidity Sensor and its Multifunctional Applications
Source: Adv Sci (Weinh). 2017 Jan 24;4(5):1600404. doi: 10.1002/advs.201600404 (PMC5441428; doi:10.1002/advs.201600404)
Supplement: Supplementary file 1 — Supplementary [file ADVS-4-na-s001.pdf]

Copyright WILEY-VCH Verlag GmbH & Co. KGaA, 69469 Weinheim, Germany, 2016.

Supporting Information

for *Adv. Sci.,* DOI: 10.1002/advs.201600404

Porous Ionic Membrane Based Flexible Humidity Sensor and its Multifunctional Applications

*Tie Li, Lianhui Li, Hongwei Sun, Yan Xu, Xuewen Wang, Hui Luo, Zheng Liu, and Ting Zhang**

Copyright WILEY-VCH Verlag GmbH & Co. KGaA, 69469 Weinheim, Germany, 2016.

Supporting Information

Porous Ionic Membrane Based Flexible Humidity Sensor and its Multifunctional Applications

*Tie Li, Lianhui Li, Hongwei Sun, Yan Xu, Xuewen Wang, Hui Luo, Zheng Liu, and Ting Zhang**

Table of Figures:

Figure S1: (a) The photograph of polymer electrolyte precursor solution. (b) The mechanical properties of PIM were carried to strain state of 383.6% by stretching machine .

Figure S2: (a) and (b) The limited response capability at low of 6.42% RH and high of 93.54% RH as well. (c) The response and recovery curves of the PIM-based humidity sensor; (d) The enlarged part of the response time. (e) The sensitivity curves of the humidity sensor for temperature range from 0 to 95 ^{o}C.

Figure S3: The stability of the humidity sensor in ambient environment tested in four days.

Figure S4: Typical SEM images of the unbroken porous structure of the PIM sample after the pressure experiment ranging from 0 to 6.8 kPa.

Figure S5: Sensitivity measurements of the PIM-based humidity sensor under (a) different mass ratios and (b) thickness.

Figure S6: Typical SEM image of the sample under the mass ratio of 6:4.

Figure S7: Typical SEM images of the synthetized samples with different thickness.

Figure S8: The Electrical Impedance Spectroscopy data of the sensor in humidity condition of dry air to 61.42 % RH with several cycles.

Figure S9: The photograph of a PDMS substrate with 10 holes; the diameter of each hole was 3 mm.

Figure S10: The relative humidity testing system.

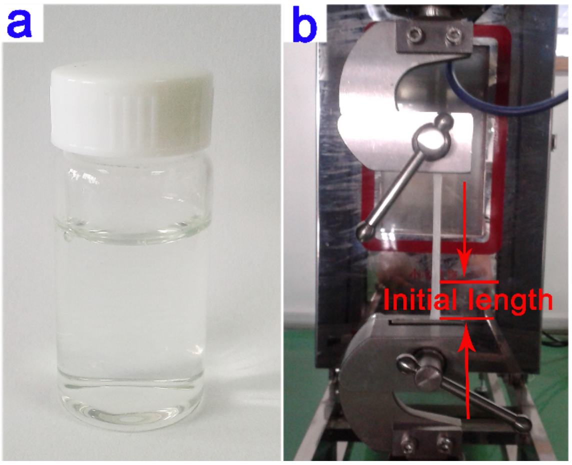

Figure S1. (a) The photograph of polymer electrolyte precursor solution that was prepared by mixing and stirring PVA and KOH in deionized water under room temperature. (b) The mechanical properties of PIM were carried to strain state of 383.6% by stretching machine .

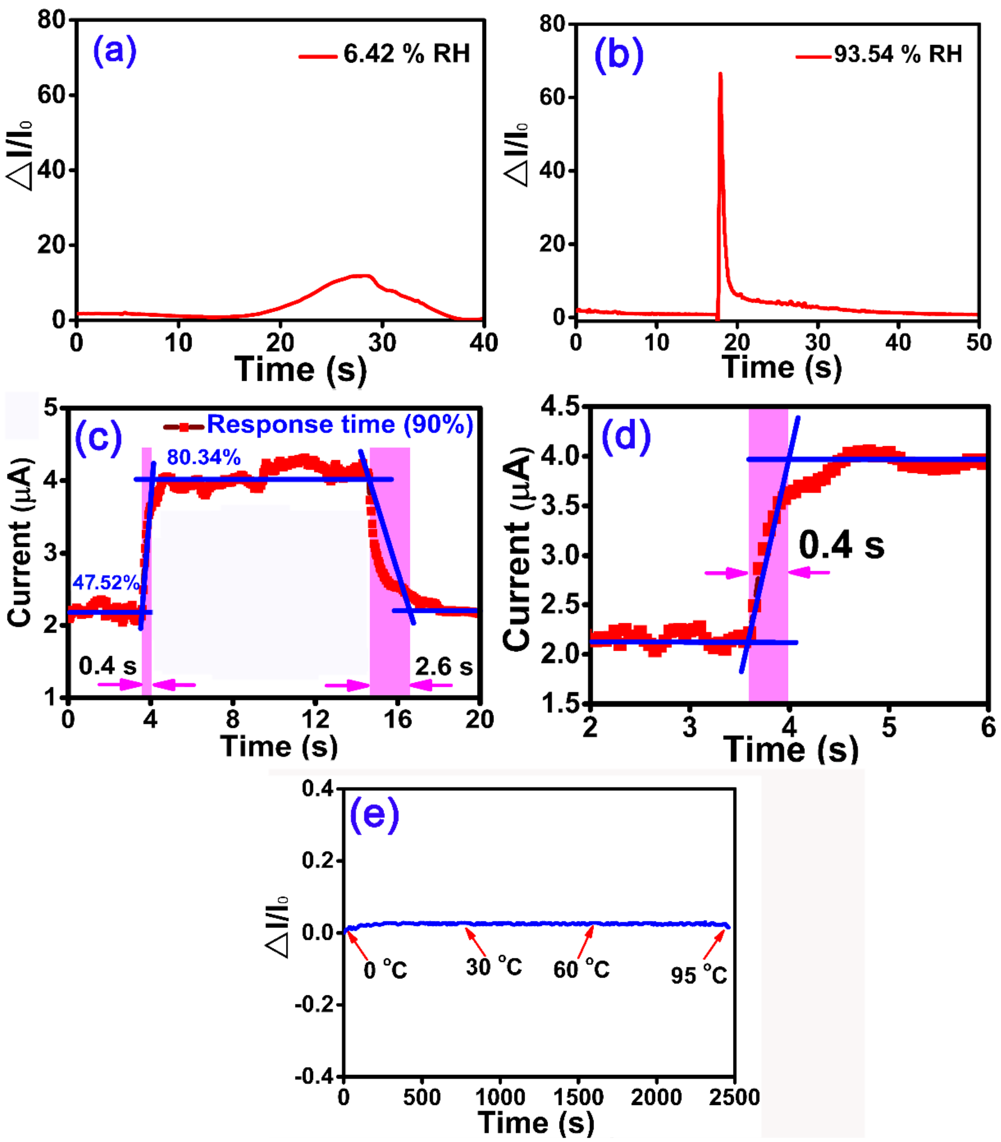

Figure S2. (a) and (b) The limited response capability at low of 6.42% RH and high of 93.54% RH as well. (c) The response and recovery curves of the PIM-based humidity sensor; (d) The enlarged part of the response time. (e) The sensitivity curves of the humidity sensor for temperature range from 0 to 95 ^{o}C.

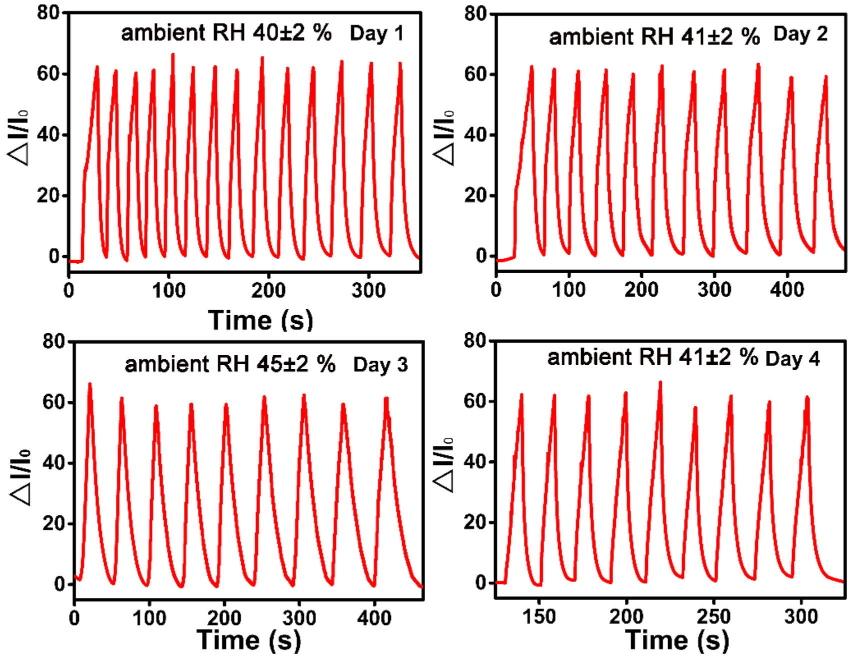

Figure S3. The stability of the humidity sensor in ambient environment tested in four days.

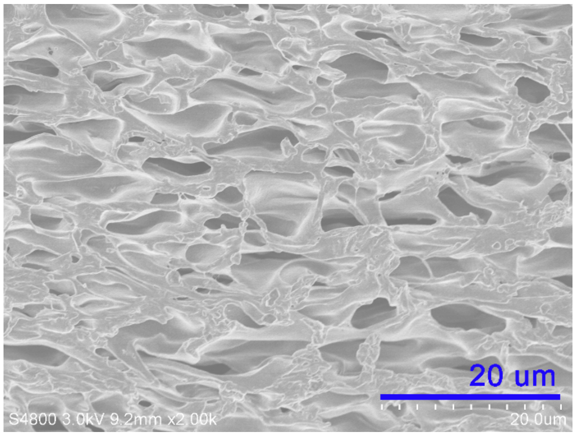

Figure S4. Typical SEM images of the unbroken porous structure of the PIM sample after the pressure experiment ranging from 0 to 6.8 kPa.

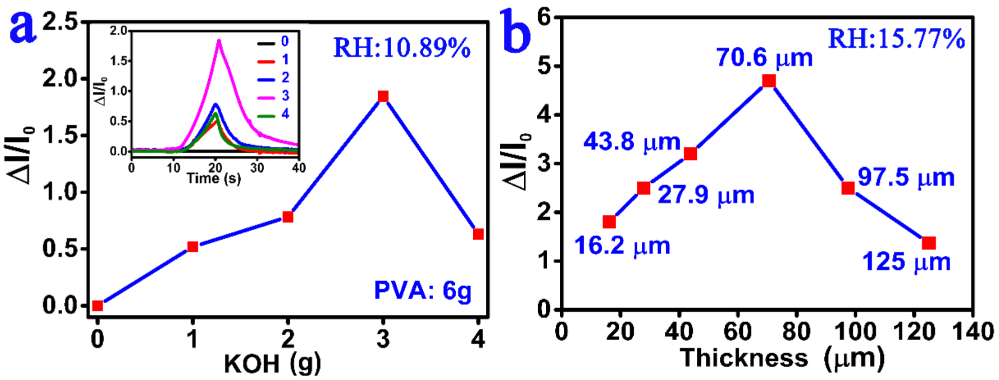

Figure S5. Sensitivity measurements of the PIM-based humidity sensor under (a) different mass ratios and (b) thickness. (The amount of PVA was 6 g)

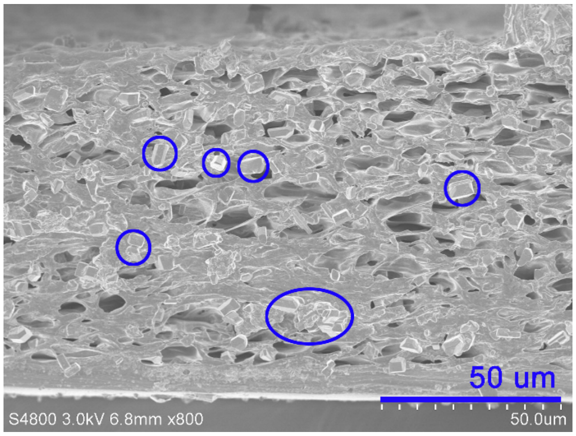

Figure S6. Typical SEM image of the sample under the mass ratio of PVA:KOH=6:4

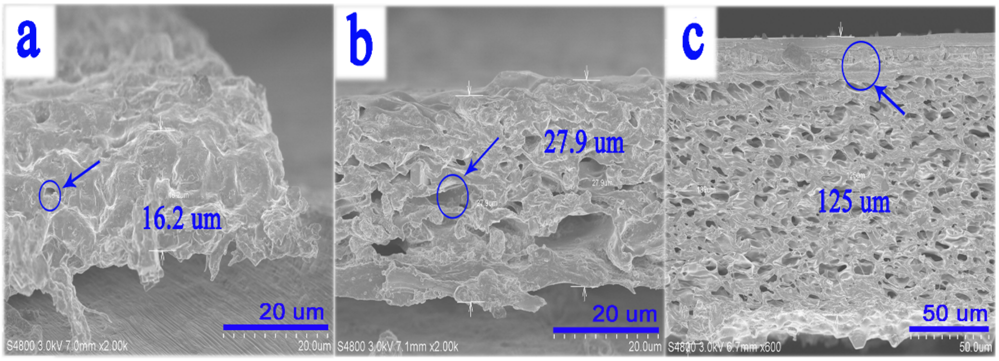

Figure S7. Typical SEM images of the synthetized samples with different thickness. (The sample was obtained at mass ratio of PVA: KOH=6:3.)

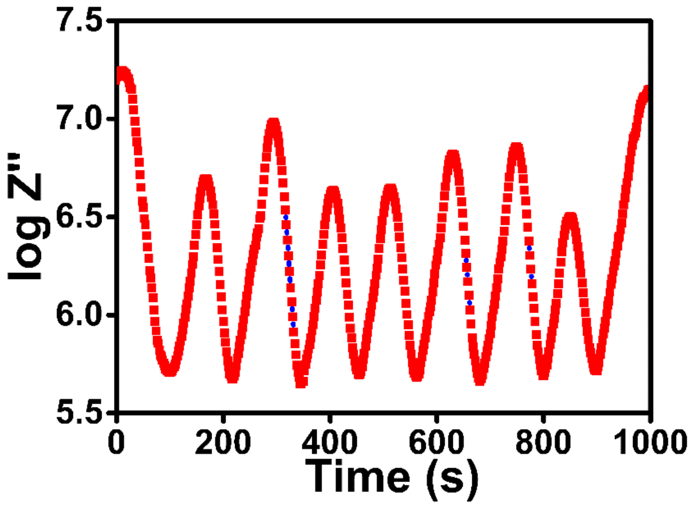

Figure S8: The Electrical Impedance Spectroscopy data of the sensor in humidity condition of dry air to 61.42 % RH with several cycles.

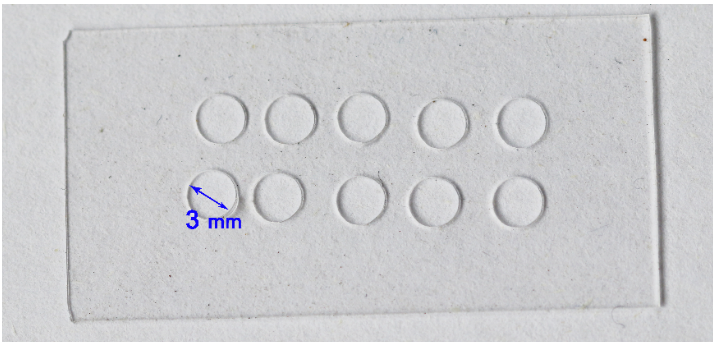

Figure S9. The photograph of a PDMS substrate with 10 holes; the diameter of each hole was 3 mm. A PIM placed on the top of a PDMS substrate can avoid directly contacting with the skin and possess a fixed distance to the measured object surface.

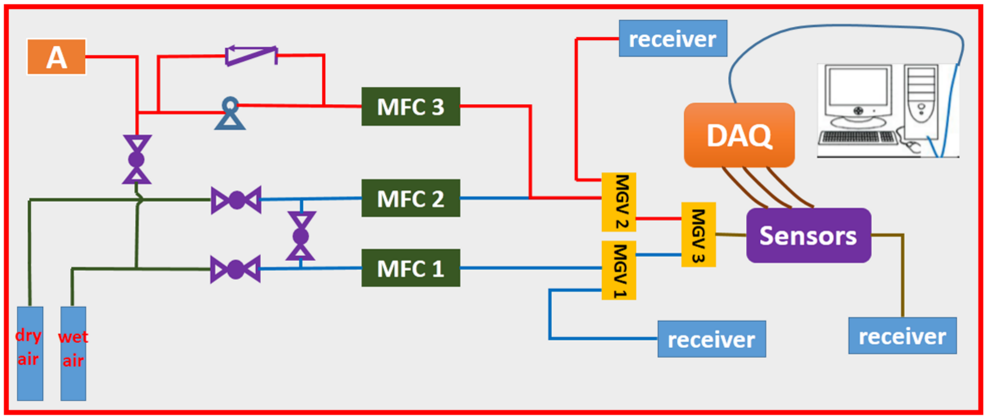

Figure S10. The relative humidity testing system.
